# Supplementary figures and images for: A national survey integrating clinical, laboratory, and WASH data to determine the typology of trachoma in Nauru
Source: PLoS Negl Trop Dis. 2022 Apr 19;16(4):e0010275. doi: 10.1371/journal.pntd.0010275 (PMC9017947; doi:10.1371/journal.pntd.0010275)

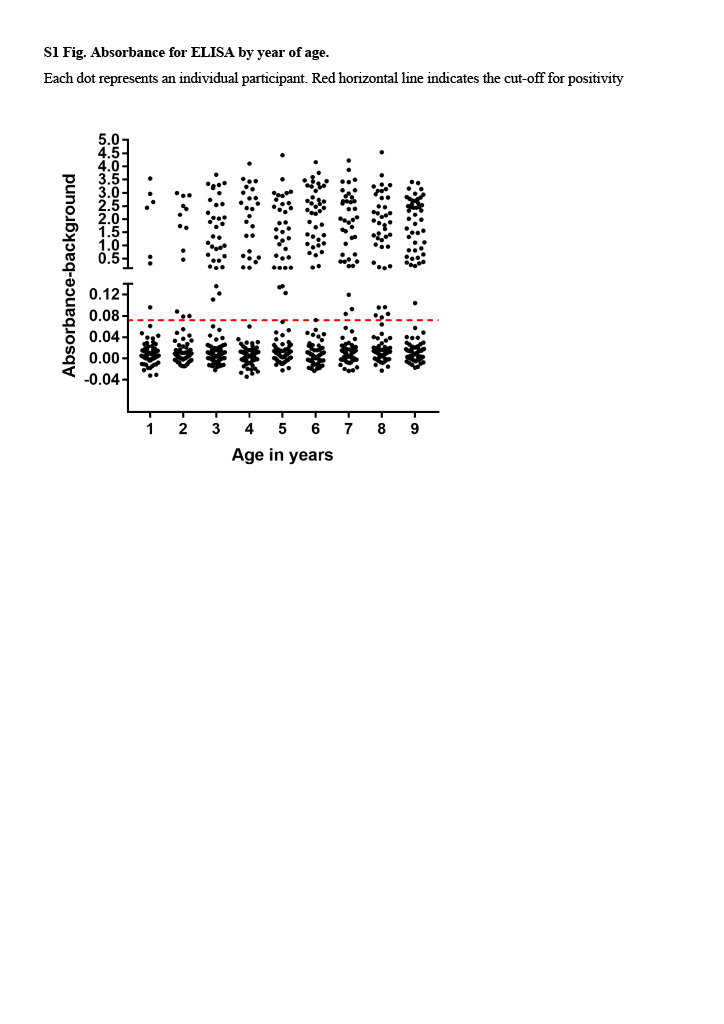

Supplement: S1 Fig — Each dot represents an individual participant. Red horizontal line indicates the cut-off for positivity. (TIF) [file pntd.0010275.s001.tif]
