# Supplementary material for: A national survey integrating clinical, laboratory, and WASH data to determine the typology of trachoma in Nauru
Source: PLoS Negl Trop Dis. 2022 Apr 19;16(4):e0010275. doi: 10.1371/journal.pntd.0010275 (PMC9017947; doi:10.1371/journal.pntd.0010275)
Supplement: S1 Table — (DOCX) [file pntd.0010275.s002.docx]

**S1 Table** **Factors associated with *Chlamydia trachomatis* positive polymerase chain reaction (PCR) in children aged 1–9 years, Nauru, July 2019 (n=780)**

| **Variable** | **n** | **PCR^+ve^**  **n (%)** | **OR (95%CI); p-value** | **aOR (95%CI); p-value^a^** |
| --- | --- | --- | --- | --- |
| **Age, increase per year** | | | | |
| 1–9 years | 780 | 272 (34.9) | 1.02 (0.95–1.09); 0.58 | 1.02 (0.96–1.10); 0.46 |
| **Gender** | | | | |
| Male | 409 | 148 (36.2) | 1.0 (reference) | 1.0 (reference) |
| Female | 371 | 124 (33.4) | 0.89 (0.67–1.17); 0.39 | 0.90 (0.66–1.23);0.51 |
| **Household source of water used for drinking** | | | | |
| Improved | 751 | 269 (35.9) | 1.0 (reference) | 1.0 (reference) |
| **Unimproved** | 22 | 3 (13.6) | 0.28 (0.18–0.44); <0.001 | **0**.**29 (0**.**19**–**0**.**44); <0**.**001** |
| Other | 7 | 0 | no events | no events |
| **Time to get drinking water** | | | | |
| Water source in the yard | 531 | 184 (34.7) | 1.0 (reference) | 1.0 (reference) |
| Travel required | 249 | 88 (35.3) | 1.03 (0.60–1.77); 0.91 | 1.25 (0.72–2.20); 0.42 |
| **Household source of water used for washing^b^** | | | | |
| Improved | 748 | 266 (35.6) | 1.0 (reference) | 1.0 (reference) |
| Unimproved | 27 | 6 (22.2) | 0.52 (0.21–1.30); 0.16 | 2.8 (0.46–17.24); 0.26 |
| **Time to get washing water** | | | | |
| Water source in the yard | 389 | 125 (32.1) | 1.0 (reference) | 1.0 (reference) |
| All face washing done at the source | 144 | 57 (39.6) | 1.38 (0.84–2.29); 0.21 | 1.34 (0.76–2.35); 0.31 |
| Travel required | 247 | 90 (36.4) | 1.21 (0.70–2.10); 0.50 | 1.35 (0.77–2.37); 0.30 |
| **Where do adults in the household usually defecate?** | | | | |
| Private latrine | 754 | 264 (35.0) | 1.0 (reference) | 1.0 (reference) |
| Other | 26 | 8 (30.8) | 0.82 (0.22–3.10); 0.78 | 0.79 (0.17–3.70); 0.77 |
| **Household latrine** | | | | |
| Improved | 693 | 249 (35.9) | 1.0 (reference) | 1.0 (reference) |
| Unimproved | 87 | 23 (26.4) | 0.64 (0.32–1.30); 0.22 | 0.89 (0.42–1.89); 0.76 |
| **Is there a functioning handwashing facility available?** | | | | |
| Handwashing available with water and with soap | 677 | 232 (34.27) | 1.0 (reference) | 1.0 (reference) |
| Handwashing available with water but without soap | 33 | 18 (54.55) | 2.3 (0.73–7.27); 0.16 | 2.21 (0.69–7.07); 0.18 |
| No functioning handwashing facility available | 67 | 22 (32.84) | 0.94 (0.27–3.24); 0.92 | 1.01 (0.30–3.42); 0.98 |

^a^ Multivariable logistic regression model adjusted for age, gender, household drinking source, availability of handwashing facility, and cluster; ^b^ Five children lived in households with access that did not fit into the improved or unimproved category; bold denotes p<0.05.
