# Supplementary material for: A national survey integrating clinical, laboratory, and WASH data to determine the typology of trachoma in Nauru
Source: PLoS Negl Trop Dis. 2022 Apr 19;16(4):e0010275. doi: 10.1371/journal.pntd.0010275 (PMC9017947; doi:10.1371/journal.pntd.0010275)
Supplement: S2 Table — (DOCX) [file pntd.0010275.s003.docx]

**S2 Table. Factors associated with antibodies against Pgp3 (demonstrated by ELISA positivity) in children aged 1–9 years, Nauru, July 2019 (n=792).**

| **Variable** | **n** | **ELISA^+ve^**  **n (%)** | **OR (95%CI); p-value** | **aOR (95%CI); p-value^a^** |
| --- | --- | --- | --- | --- |
| **Age, increase per year** | | | | |
| 1–9 years | 792 | 276 (34.9) | 1.23 (1.14–1.32); <0.001 | **1**.**23 (1**.**15**–**1**.**32); <0**.**001** |
| **Gender** | | | | |
| Male | 416 | 142 (34.1) | 1.0 (reference) | 1.0 (reference) |
| Female | 376 | 134 (35.6) | 1.07 (0.85–1.34); 0.57 | 1.09 (0.85–1.40); 0.51 |
| **Household source of water used for drinking** | | | | |
| Improved | 763 | 265 (34.7) | 1.0 (reference) | 1.0 (reference) |
| Unimproved | 22 | 10 (45.5) | 1.57 (0.80,3.07); 0.19 | **1**.**70 (1**.**02,2**.**86); 0**.**04** |
| Other |  | 1 (14.3) | 0.31 (0.03–3.21); 0.33 | 0.23 (0.03–1.64); 0.14 |
| **Time to get drinking water** | | | | |
| Water source in the yard | 537 | 197 (36.7) | 1.0 (reference) | 1.0 (reference) |
| Travel required | 255 | 79 (31.0) | 0.77 (0.51–1.17); 0.22 | 0.74 (0.47–1.18); 0.21 |
| **Household source of water used for washing^b^** | | | | |
| Improved | 759 | 263 (34.7) | 1.0 (reference) | 1.0 (reference) |
| Unimproved | 28 | 13 (46.4) | 1.63 (0.72–3.69); 0.24 | 1.85 (0.92–3.71); 0.08 |
| **Time to get washing water** | | | | |
| Water source in the yard | 391 | 140(35.8) | 1.0 (reference) | 1.0 (reference) |
| All face washing done at the source | 148 | 58(39.2) | 1.16 (0.72–1.85); 0.55 | 1.03 (0.64–1.66); 0.90 |
| Travel required | 253 | 78(30.8) | 0.80 (0.51–1.25); 0.32 | 0.80 (0.48–1.32); 0.38 |
| **Where do adults in the household usually defecate?** | | | | |
| Private latrine | 762 | 264(34.7) | 1.0 (reference) | 1.0 (reference) |
| Other | 30 | 12(40.00) | 1.26 (0.48–3.29); 0.64 | 0.74 (0.30–1.84); 0.52 |
| **Household latrine** | | | | |
| Improved | 704 | 246(34.9) | 1.0 (reference) | 1.0 (reference) |
| Unimproved | 88 | 30 (34.1) | 0.96 (0.58–1.59); 0.88 | 0.92(0.55–1.55); 0.75 |
| **Is there a functioning handwashing facility available?** | | | | |
| Handwashing available with water and with soap | 689 | 233 (33.82) | 1.0 (reference) | 1.0 (reference) |
| **Handwashing available with water but without soap** | 34 | 18 (52.94) | 2.20 (0.96–5.03); 0.06 | **2**.**49 (1**.**00**–**6**.**19); 0**.**05** |
| No functioning handwashing facility available | 66 | 22 (33.33) | 0.98 (0.42–2.25); 0.96 | 1.0 (0.44–2.27); 1.0 |

^a^ Multivariable logistic regression model adjusted for age, gender, availability of handwashing facility, and cluster; ^b^ Five children lived in households with access that did not fit into the improved or unimproved category; bold denotes p<0.05.
