# Supplementary material for: A national survey integrating clinical, laboratory, and WASH data to determine the typology of trachoma in Nauru
Source: PLoS Negl Trop Dis. 2022 Apr 19;16(4):e0010275. doi: 10.1371/journal.pntd.0010275 (PMC9017947; doi:10.1371/journal.pntd.0010275)
Supplement: S1 Text — (DOC) [file pntd.0010275.s004.doc]

A national survey integrating clinical, laboratory, and WASH data to determine the typology of trachoma in Nauru

1. **Methods**
   1. **Serological testing for** **anti-Pgp3 antibodies****: Enzyme-linked immunosorbent assay**

DBS samples and dried serum spot (DSS) standards were tested in duplicate. DSS positive for anti-Pgp3 antibodies across a range of responses, as well as negative human serum (NHS)[1], were run on each plate. 6mm discs were eluted in 250μL phosphate buffered saline + 3% Tween20 (PBST) and 5% skim milk powder in a 96-well flat bottom plate, overnight at 2–8°C. 50μL of each sample was pipetted into Pgp3-coated 96-well plates, incubated 2h at room temperature, washed 4 times with 200μL PBST, then incubated 1h at room temperature (RT) with 50μL horseradish peroxidase conjugate. After a final wash, the reaction was developed with 50μL of tetramethylbenzidine in darkness for 26min at RT, then stopped using 50μL 1M H_2_SO_4_. Optical density was measured at 450nm on a Sunrise plate reader (Tecan Group Ltd, Switzerland). The average result from 6 blank wells was subtracted from each averaged DBS and DSS absorbance value, and the result normalised against the mid-range DSS positive control to account for inter-plate variation.[2] Plates were acceptable if results for 3 out of the 4 positive controls and the NHS control fell within pre-determined ranges (established during assay validation) and the positive controls were at least 50 times higher than background.

# References

1. Gwyn S, Cooley G, Goodhew B, et al. Comparison of Platforms for Testing Antibody Responses against the Chlamydia trachomatis Antigen Pgp3. *The American journal of tropical medicine and hygiene* 2017; **97**(6): 1662-8.

2. Migchelsen S, Martin D, Southisombath K, et al. Defining Seropositivity Thresholds for Use in Trachoma Elimination Studies. *PLoS Neglected Tropical Diseases* 2017.
