# Supplementary material for: A national survey integrating clinical, laboratory, and WASH data to determine the typology of trachoma in Nauru
Source: PLoS Negl Trop Dis. 2022 Apr 19;16(4):e0010275. doi: 10.1371/journal.pntd.0010275 (PMC9017947; doi:10.1371/journal.pntd.0010275)
Supplement: Attachment — Submitted filename: Response to reviewers.docx [file pntd.0010275.s005.docx]

**RESPONSES TO REVIEWERS’ COMMENTS - PLOS NTD**

**A national survey integrating clinical, laboratory, and WASH data to determine the typology of trachoma in Nauru**

Please note, line numbers used in this document refer to the tracked changes version of the manuscript. We have noted the line numbers changed for different users of Word, so have provided a pdf document for which the line numbers referred to in the response document are accurate.

**METHODS**

**Reviewer #1: This was no doubt a well-planned study and expensive to undertake. I don't understand why the WHO Simplified grading was used instead of a finer grading of active trachoma. If one is going to spend the time, the effort and the money to do PCR and serology, one would expect to at least grade trachoma with some precision. The Simplified grading scheme was designed for health workers to assess the public health impact of trachoma. To determine if the SAFE strategy or even MDA was needed in Nauru on could always also categorise the findings from a more specific gradings into TF or TT. It is highly likely that many children will have had F1 or F2 but not sufficient large follicles to be TF and equally many will have had C1 or C2 without reaching the threshold for TS!!**

This comment has prompted us to recognise that the manuscript does not adequately identify that the clinical assessment component of this work was a population-based prevalence survey conducted for programmatic purposes, to assess the need for interventions under WHO guidelines. We were fortunate to be able to add the laboratory research components, which we believe greatly added to overall usefulness. Even if we had collected more detailed clinical information, for example using the FPC system, we would have still needed to compare against the simplified system which is the standard for programmatic decisions made globally. In response to the reviewer’s comment, we have attempted to better differentiate between the population-based clinical prevalence work and the additional laboratory research components, and updated the manuscript as follows:

- line 149 - “The population-based prevalence survey was undertaken by the Nauru Ministry of Health with the support of the Fred Hollows Foundation using standard Tropical Data methods.”
- line 203 - “The research component was undertaken alongside the national survey to collect information on laboratory correlates of clinical findings. Consent from carers was sought for individuals aged 1–9 years of age who were participating in the national survey.”
- line 273 - “Results of the laboratory testing were linked to the clinical results of the national survey and analysed together.”

**For a very detailed research study like this, one wonders why were photos of the tarsal plate not taken?**

As noted above, the prevalence survey for trachoma used standard, WHO-endorsed Tropical Data methods which do not include collection of photographic data. Given the ongoing discussion about how to take high quality photographs of relevant parts of the eye, they have not been adopted as standard practice in either research or programmatic contexts.

**Again if the challenge was to try to infer something about the history or trachoma in Nauru more detail should be provided about the findings in each of the age groups over 9 years of age.**

We have provided more detail on TF and TI in older age groups. We have also broken down the trichiasis findings by age and gender. Changes can be found at the following lines:

- 318: Of 317 children aged 10–14 years, 113 (35.6%) had TF and 10 (3.2%) had TI.
- 327: “All six cases were unilateral. Five of the cases were in individuals aged 15–39 years (60% male), and one in a female participant aged ≥ 40 years”.
- 332: “In individuals aged ≥15 years, 5.1% (70/1380) had TF and 0.5% (7/1380) had TI.”

**Although WASH data were collected for the households, no data are presented for facial cleanliness. This is in many ways the key indicator for personal hygiene. it is very surprising that this was left out.**

Like trachoma surveys conducted around the world, the Nauru national survey used standard Tropical Data methods which do not include collection of data on facial cleanliness.

**When a case was found the child was given 2 tubes of tetracycline ointment. Why wasn't the household treated with azithromycin or was MDA done later?**

We gave tetracycline eye ointment to presumed cases of active trachoma as per standard Tropical Data methods, which recommended tetracycline eye ointment or azithromycin be given to subjects found to have active trachoma or presumed bacterial conjunctivitis. Following the survey, the Nauru Ministry of Health undertook a nationwide antibiotic MDA which was completed in March 2020.

We have updated the manuscript at line 507 to make it clear that azithromycin MDA was to be undertaken. It now reads … “Nauru implemented the SAFE strategy, including azithromycin mass drug administration for elimination of trachoma as a public health problem.”

**Reviewer #2: (No Response)**

**Reviewer #3: Generally, the methods are described adequately, with the exception of methods to detect and manage contamination in the field and laboratory. These need to be expanded as described below**

Reviewer 3 has mentioned the potential for contamination in a number of comments, and we take this opportunity to respond to all of them here.

Under our standard operating procedures, we implemented rigorous control measures to prevent contamination in both the field and laboratory. In the field when collecting conjunctival specimens, we ensured the swab head was only in contact with an individual’s conjunctiva, and graders cleaned their hands with alcohol-based hand gel between each subject. The staff member collecting the DBS wore gloves, which were changed and hand hygiene performed between each subject.

The serology testing on dried blood spots used methods to detect and manage contamination based on adherence to the standard operating procedure prepared by the US Centers for Disease Control and Prevention (CDC), which guided our assay validation and testing. For DBS a blank spot was punched before each new patient spot to reduce the risk of carry over. In addition, the testing involved use of ELISA, involving5 standards with pre-determined ranges that needed to be met, and a requirement that its signal to noise ratio exceeded 50. The quality control criteria required to pass each testing plate followed CDC Instructions for Use. Additionally, the aliquoting of reagents and samples was performed in a biological safety class hood class II, which was decontaminated daily with daily prepared 1% bleach, followed by 80% ethanol. Pipette tips were discarded after single use. PCR adhesive plate sealers were used to seal the plates for elution and in-between incubation steps, which were all performed at room temperature inside a biological safety hood class II. Gloves were frequently changed and laboratory gowns and protective eye wear worn at all times. Workflow was uni-directional. Work and bench spaces were decontaminated daily with daily prepared 1% bleach and 80% ethanol. Good laboratory practice was maintained, and biological waste disposed of according to safe laboratory procedure.

For PCR testing of swabs, separate and physically distinct areas in the laboratory were designated for sample preparation, DNA extraction and PCR amplification. Each sample was uncapped in a biological safety hood class II, placed on board racks with the collection swab remaining inside the tube, then directly placed onboard the Roche COBAS 6800 instrument. Gloves were changed in between handling test reagents and samples. Testing was according to standard operating procedures and manufacturer’s Instructions for Use.

The assay reagents included uracil-n-glycoslyase, which prevents carry over contamination onboard the instrument. The target and DNA-IC sequences were amplified simultaneously utilizing a universal PCR amplification profile with predefined temperature steps and number of cycles. The master mix includes deoxyuridine triphosphate (dUTP), instead of deoxythimidine triphosphate (dTTP), which is incorporated into the newly synthesized DNA (amplicon). Any contaminating amplicon from previous PCR runs are eliminated by the AmpErase enzyme, which is included in the PCR master mix, during the first thermal cycling step.

In addition, a schedule of environmental monitoring is performed using multiple sterile saline swabs, which are wiped over the surfaces in the various laboratory areas used for sample preparation, extraction, and PCR amplification. All surfaces, benchtops and biological safety hoods class II are decontaminated daily with daily prepared 1% bleach and 80% ethanol. Uni-directional workflow was maintained, gloves frequently changed with laboratory gowns and protective eye wear worn at all times. Good laboratory practice was maintained, and biological waste disposed of according to safe laboratory procedure.

We have now provided additional detail in the manuscript to describe measures taken to prevent contamination. These can be found at the following lines:

- 211: “Control procedures were implemented to avoid cross contamination of specimens in the field. We took care to ensure the swab head was only in contact with an individual’s conjunctiva, and graders cleaned their hands with alcohol-based hand gel between each subject.”
- 218: The staff member collecting the DBS wore gloves, which were changed, and hand hygiene performed, after each subject.
- 234: We implemented rigorous control measures to prevent and identify contamination in the laboratory. There were separate and physically distinct areas in the laboratory designated for sample preparation, DNA extraction and PCR amplification. Each sample was uncapped in a biological safety hood class II, placed on board racks with the collection swab remaining inside the tube, then directly placed onboard the Roche COBAS 6800 instrument. Gloves were changed in between handling test reagents and samples. Testing was performed according to standard operating procedures (SOPs) and manufacturer’s Instructions for Use. The assay reagents include uracil-n-glycoslyase, which prevents carry over contamination onboard the instrument. Any contaminating amplicon from previous PCR runs are eliminated by the AmpErase enzyme, which is included in the PCR master mix, during the first thermal cycling step.
- 250: A blank spot was punched after each patient spot to reduce the risk of carry over contamination. The quality control criteria required to pass each testing plate followed CDC Instructions for Use

We believe that our findings are entirely consistent with the hypothesis that the high prevalence of ocular *C. trachomatis* has led to a high prevalence of TF in Nauruan children. Reviewer 3 concludes: “…so the likelihood that the infection data supports the finding of TF is strong.” We agree, and note that our age-specific findings on TF prevalence, ocular *C. trachomatis* detection, and seropositivity are internally consistent and do not require contamination as an explanation.

***Given that the infection rate is so high, higher than the rate of TF, and does not go up with age, suggests that there may have been field and/or lab contamination***. We do not agree with this conclusion. Seropositivity is a result of cumulative exposure to *C. trachomatis* and indeed goes up rapidly with age throughout childhood years in trachoma endemic settings, as has been well established in the literature and as seen in our data. The relatively flat shape of the conjunctival *C. trachomatis* infection versus age curve that we report is also entirely consistent with data from other settings, for example the three trachoma-endemic populations described in detail by Solomon et al. (2003).[1]

***Were control swabs taken to check for field contamination?*** We acknowledge that others have used air-exposed control swabs, but this is by no means a universal practice, and may also be portrayed as all-too-obvious alert to the field team that such samples must be particularly carefully safeguarded, so by design cannot undertake the function they purport to fulfil. Air-exposed control swabs have not been used in many other published trachoma studies including those performed in Papua New Guinea, Kiritimati Island, Kiribati, Tanzania, and The Gambia.[1-4] The control procedures we implemented are consistent with those described by Solomon et al. (2003) [1]

***The high rate of infection positivity in those without disease is further concerning*** and in a separate comment, ***when fully 61% of positive test of infection is not accompanied by presence of disease, and half of those don’t even have positive serology, then one must investigate if contamination is an issue.*** We expect no disease but positive serology, given the cumulative nature of seropositivity-but that does not hold for infection.” Again, we do not believe that this is a correct interpretation of our findings. First of all, the absence of TF is not the same thing as no disease. In the three sites reported by Solomon et al. (2003), 68% (Jareng, The Gambia), 39% (Kongwa, Tanzania) and 34% (Rombo, Tanzania) had neither TF nor TI.[1] In trachoma endemic settings, PCR positivity is more likely to be accompanied by seropositivity and clinical findings in older children, as cumulative exposure and conjunctival damage increases. This expected epidemiological picture is precisely what we report: seropositivity which increases by year of age (9%–48%) and TF prevalence which increases steadily in children aged 1–6 years (3%–37%). Our data on increasing TI across childhood provide further evidence: TI was observed in 0.3% (1/346) of children <4 years of age, 1.7% (8/472) of children aged 5–9-years, and 3.2% (10/317) of children aged 10–14 years. In response to this comment, we have updated the manuscript to include additional detail about TI prevalence. For completeness we have also included information on TF prevalence in children aged 10–14 years. These amendments can be found at the following lines:

- 318: “Of 317 children aged 10–14 years, 35.6% (113) had TF”
- 322: “TI prevalence increased with age, from 0.3% (1/346) of children <4 years, 1.7% (8/472) of children aged 5–9-years, to 35.6% (113/317) of those aged 10–14 years”

We acknowledge that contamination is always a risk in PCR-based studies, which is why we used comprehensive field and laboratory procedures to avoid it. Given the internal consistency of our findings, and their consistency with reports from other trachoma-endemic settings, we see no reason for contamination to be seriously discussed as a contributor to our findings.

**While the sample size was done to provide estimates of TF, since further analyses were done with WASH variables, the authors should provide calculations of power to detect differences when rates of access to some variables were so high**

We have updated the manuscript at line 282: “A post-hoc power calculation was undertaken to investigate the detectable alternative for the association between a two-category WASH variable and TF. For 818 children, assuming the variable had an 80:20 split (e.g. 654 in one category vs 164 in the other) and there was 25% TF in the reference WASH group, we had 80% power to detect a statistically significant difference if the percentage of TF in the comparator WASH group was 36% or greater.”

**RESULTS**

**Reviewer #1: The data for adults should be presented appropriately broken down by age and gender.**

We have updated the manuscript at line 327: “There were six (0.4%) cases of trichiasis identified among 1,380 participants aged ≥15 years. All six cases were unilateral. Five of these cases were in individuals aged 15–39 years (60% male), and one in a female participant aged ≥40 years.”

**The finding of one child under 10 with trichiasis is most unusual. This should be further explained as should the one case in those 10 to 15.**

We have provided the specific age and sex of each case in the text at line 328: “There were two cases in children, a 9-year-old [male] and a 13-year-old [female] ([Fig 1]). Both cases were unilateral.” In trachoma hyperendemic settings in Africa, particularly Ethiopia, paediatric TT is not unheard of, though it remains a rare exception.

**Reviewer #2: (No Response)**

**Reviewer #3: Table S1 should really be a table in the text itself**

We have removed the table from the supplementary material and included it as Table 1 in the text.

**CONCLUSIONS**

**Reviewer #1: The rates of TF are low in the children less that 5 years, although the PCR rates are high. Is this because these** **children had active trachoma but less that the 5 follicles 0.5mm needed to be called TF?**

As noted above, the clinical survey was performed using standard Tropical Data methods. These methods use the WHO simplified grading tool for clinical assessment, which does not include use of a fine grading system. We therefore cannot comment on the number of follicles in children who did not meet the criteria for TF.

**With the suggestion the "TS may not have been graded accurately" and the lack of a more detailed grading of tarsal scarring or photo documentation, these findings are really just speculation.**

While grading of TS is included in the standard Tropical Data training system, (along with TF, TI and TT) field based inter-grader agreement testing is only done on TF, recognising the additional difficulty of assessing TS. As the prevalence survey used standard Tropical Data methods, this is a limitation shared by all standardised trachoma surveys.

We have added further details about the grader training to the manuscript at the following lines:

- 149: “The population-based prevalence survey was undertaken by the Nauru Ministry of Health with the support of the Fred Hollows Foundation using standard Tropical Data methods. Tropical Data uses the same systems and methods as the Global Trachoma Mapping Project.”
- 190: “Prior to conducting the survey, graders and recorders were required to pass internationally standardised training using the Tropical Data system.”

Having reflected on the reviewer’s feedback we have removed reference to difficulties with TS grading from this section and incorporated it into the paragraph on limitations, at line 487: “An additional limitation shared by all standardised prevalence surveys using the Tropical Data method is that graders are trained to assess TS as “easily visible scarring” as per the WHO simplified grading system definition. This encompasses moderate to severe scarring and it is possible some mild scarring may be missed.”

**The "trachoma paradox" in the Pacific is a somewhat fanciful construct. It does not take into consideration that the rates and severity of trachoma can change over time. With population growth and increased crowding, without commensurate improvements in personal and community hygiene, trachoma can become more common and more severe.**

We cannot agree with the Reviewer's suggesiton that the “trachoma paradox” is a fanciful construct.

The empirical evidence underpinning the concept has been gathered during extensive research undertaken to better understand the epidemiology of trachoma in the Pacific. This includes a series of detailed, published investigations in the Solomon Islands, Vanuatu, PNG, and Kiribati. [3, 5-11]

Several of these studies have demonstrated that in evaluation units with a moderate to high baseline prevalence of TF, there is a low prevalence of ocular *C. trachomatis* infection in 1–9-year-olds, low prevalence of serological markers of previous *C. trachomatis* infection in 1–9-year-olds, and low prevalence of conjunctival scarring, superior limbal pannus, and Herbert’s pits in 10–14-year-olds.

We agree with the Reviewer that rates and severity of trachoma can change over time, but do not see how such changes can explain the similarity of well-documented findings across multiple Pacific Island nations.

**The term "paratrachoma" was used by some for a while in the 1960s, as a term for chlamydial conjunctive infection that after a period of time resolved on its own without blinding sequelae. It seems this term has been misinterpreted. Paratrachoma was another name for “inclusion conjunctivitis”. It could be caused by a single infection with any serovar. It was commonly seen with the genital strains because there was usually only a single infective episode. The person was not subjected to the repeated episodes of reinfection required for the development of “trachoma”. The suggestion that these children, 24% of them, all had an almost simultaneous single infection seems just wrong.**

We agree the term paratrachoma is used variably in the literature, but we do not believe we have misinterpreted it.

Nauru has a high prevalence of sexually transmitted infections, including *C. trachomatis*.[12] Ocular infection with UGT strains of *C. trachomatis* can cause an acute follicular conjunctivitis indistinguishable from trachoma.[13, 14] It is possible that this phenomenon may be contributing to apparent TF prevalence in Nauru.

Consequently, at line 453 we have suggested that further research investigating the contribution of specific *C. trachomatis* serovars to eye infection in Nauru via detailed molecular epidemiology of both ocular and genital *C. trachomatis* is required.

We have updated the manuscript at line 448 to state: “Another possibility is that some of the TF, *C. trachomatis* and antibody positivity may be due to inclusion conjunctivitis, caused by ocular infection with urogenital strains of *C. trachomatis*”

**Reviewer #2: (No Response)**

**Reviewer #3: The limitations in the discussion need to be expanded, and the conclusion around infection need to be re thought if there is in fact evidence of contamination.**

In response we have substantially amended the discussion of limitations to include interpretation of Pgp3 antibodies, assessment of trachomatous scarring, and suggestions for additional WASH variables which could be collected in settings with widespread access to water and sanitation facilities.

Please refer to our earlier more detailed response to the comments about contamination.

In regard to the other issues, lines 479–498 now read: “A number of limitations must be acknowledged. Firstly, PCR data on infection were missing for 4.6% of participants and serology missing for 3.2%. Second, as Pgp3 antibodies do not differentiate exposure to ocular from genital strains of chlamydia, seropositivity in an individual could reflect a range of potential exposures including trachoma, inclusion conjunctivitis, and perinatal transmission during childbirth. An additional limitation shared by all standardised prevalence surveys using the Tropical Data method is that graders are trained to assess TS as “easily visible scarring” as per the WHO simplified grading system definition. This encompasses moderate to severe scarring, and it is possible some mild scarring may be missed. Finally, WASH variables collected under Tropical Data methods were developed for settings where there is poor access to sanitation and water. In settings such as Nauru, where there is widespread access to safe household sanitation and water, the collection of additional variables associated with trachoma such as household crowding, and the frequency of face and hand washing should be considered.”

**EDITORIAL AND DATA PRESENTATION MODIFICATIONS?**

**Reviewer #1: Data for the adults needs to be presented more fully.**

**If photos were taken of clean face data collected, they need to be presented.**

As the national prevalence survey used standard Tropical Data methods, no photographic data was collected.

We have provided more detail for adults. Changes can be found at line:

- 327: “All six cases were unilateral. Five of the cases were in individuals aged 15–39 years (60% male), and one in a female participant aged ≥ 40 years.”
- 332: “In individuals aged ≥15 years, 5.1% (70/1380) had TF and 0.5% (7/1380) had TI.

**Reviewer #2: (No Response**

**Reviewer #3: There needs to be much more clarity on how the potential for contamination of samples was addressed in the field and in the lab. This is a major concern.**

Please refer to our earlier detailed response to comments about contamination.

**The authors also have to use TT in place of trichiasis in several places, and by the results, they have to decide if they believe the TS data and what they found is not TT , or they don't and it is TT. The ramifications of either decision deserve further discussion.**

We believe the TS data. Six cases of trichiasis were identified, and eye nurses who had been trained to recognize TS and were provided with 2.5× magnifying loupes to help identify it reported specifically for each of them that TS was absent. That does not rule out minor degrees of conjunctival scarring that would be insufficient to confer a diagnosis of TS according to the simplified system, as we note in the limitations section of the paper, but such scarring should be insufficient, by definition to produce trachomatous trichiasis. For this reason, we are of the view that “TT” should not be used to label the trichiasis cases identified in this study.

We have updated the manuscript at line 331 to state: “It was reported specifically by the graders that no individual with trichiasis has TS”

**SUMMARY AND GENERAL COMMENTS**

**Reviewer #1: This is an experienced group, working on a small island, using some very sophisticated tests. However, they seem to have omitted the key elements required for the sort of study they are trying to present. If the study were just a rapid assessment of trachoma, the PCR and serology are not needed. If this were an attempt to study the dynamics and kinetics of trachoma in Nauru, then they must use a finer grading system for trachoma and also present the data for the adults. If this were an attempt to look at the reasons trachoma might occur in Nauru, then in addition to the WASH factors, they must include facial cleanliness.**

Following the Reviewer’s logic, one could only ever conduct two types of trachoma assessments: one, a basic survey using the Tropical Data surveillance method or similar with no further embellishment, or one with a highly detailed clinical assessment for all individuals, complemented by PCR and dried blood spot testing. In fact, there have been multiple publications reporting hybrid studies that used essentially the same approach that we have adopted, combining a laboratory-based “research” component with a standard survey based on methods of the Global Trachoma Mapping Project or, more recently, Tropical Data. For example, Papua New Guinea, Solomon Islands, Vanuatu, Kiribati, and Fiji [2, 3, 8, 10, 15]. We believe our findings are internally consistent and robust and are an important addition to understanding trachoma epidemiology in the region.

We agree with the Reviewer that collecting information on facial cleanliness would have been interesting, its absence in no way diminishes our assessment of the data we have.

**Reviewer #2: This is a well written an interesting study evaluating active trachoma, *C. trachomatis*, and serologic markers of *C. trachomatis* in Nauru. These data are interesting given the ongoing investigation of the “Pacific enigma”.**

**Were WASH facilities observed or data collected by participant report only? (These may appear in the cited paper but a sentence so that readers don’t have to refer to the cited paper would be helpful).**

We have updated the manuscript at line 177: … “data on handwashing facilities and access to toilet/latrine (if shared) were collected through head of household report.”

**How many household WASH indicators were entered into stepwise models and were these variables possibly collinear? Stepwise regression models can be a little problematic in that they tend to overfit, with over-estimated effect sizes and under-estimated variances. Depending on how many candidate variables are included (which isn’t clear from the methods), could all of the candidate variables be included (assuming there aren’t major problems with collinearity)? Looking at the supplemental tables, it’s a little unclear if a stepwise procedure was actually used (looks like all candidate variables are in the models), so if no stepwise procedure was used I would update the methods accordingly.**

We have updated the manuscript at line 279 to state: “Logistic regression analyses were used to assess relationships between these outcomes and age, gender, and household-level WASH variables. First univariable analyses were undertaken, and then multivariable analyses with age, gender, time to get drinking water, time to get washing water, and latrine classification included as covariables.”

**Page 20, Line 347 – could the authors speculate as to why they’re observing a lower seropositivity among PCR positives in their study versus Kiribati?**

It is important to acknowledge methodological differences between our study and the study in Kiribati.[3] In Nauru, we aimed to take a swab for PCR testing from every child enrolled in the study whereas in Kiribati, swabs were only taken from children found to have active trachoma (TF/TI) and from a two-thirds sample of children without these signs.[3]

Different patterns of seropositivity and PCR positivity may be due to intensity of exposure by age. Comparison of our seropositivity data with data from Kiribati suggests some difference in the pattern of exposure between the two settings. This is particularly apparent in the 1–4-years age group: in Nauru there is a gradual increase in seropositivity by year (9–29%) whereas in Kiribati there is a sharper increase (7–58%) with large increase between one-year-olds (8%) and 2-year-olds (33%).

Our finding of a gradual increase in seropositivity with age within the 1–9-year-old age group in Nauru fits with our interpretation of the setting stated at line 504: … “our results show trachoma is rife in Nauruan children and suggest there has been a recent and substantial introduction of ocular *C. trachomatis*. The absence of TS and TT in adults further supports our conclusions.”

**Are there plans for antibiotic-based intervention for trachoma in Nauru (or, do the authors believe its warranted given these results)?**

Yes, the prevalence survey and research activities led to the conclusion that trachoma was a substantial public health problem and the Nauru Ministry of Health undertook a nationwide azithromycin MDA in March 2020.

We have updated the manuscript at line 506 to state: “Based on these findings, Nauru implemented the SAFE strategy, including azithromycin mass drug administration for elimination of trachoma as a public health problem”

**The relationship between WASH and trachoma variables could very well be due to unmeasured confounding, for example, by SES (in previous studies and in this one). It would be worth mentioning that cross-sectional associations shouldn’t infer causality and that there’s high risk of unmeasured confounding here. I think further survey work won’t really help us understand these questions, but randomized controlled trials might.**

We have removed the recommendation for further survey work and updated the manuscript at line 470 to state “…or it may be due to an unmeasured confounding variable, such as socioeconomic status”.

**Reviewer #3: The manuscript adds data on trachoma in the Pacific Islands and supports a more recent introduction of trachoma. My main concern is the possible presence of contamination in the infection results. Control measures in the filed or the lab are not described, and the results are strongly suggestive of a problem. That said, there needs to be infection in order to have contamination, so the likelihood that the infection data supports the finding of TF is strong. Nevertheless, this concern is given scant attention and needs to be addressed as described below**

See detailed response above to comments about contamination.

**Abstract: the conclusion that “the absence of trichiasis with trachomatous scarring suggest a relatively recent increase in transmission intensity” is liable to be confusing to readers who are unfamiliar with the distinction between trachomatous trichiasis (TT) and trichiasis due to other causes, especially as the abstract presented results on the prevalence of trichiasis as though it was TT, and thus were given importance like the rest of the trachoma related data. Either the authors believe their TS data and this is likely not trachomatous trichiasis, or they do not and the prevalence can be reported as TT. The sentence does need to be modified. Perhaps if that sentence on prevalence of trichiasis were deleted from the abstract, and a statement that although trichiasis was found, the absence of scarring in any of the cases suggested it may not be due to trachoma. Then the conclusion would make more sense to the reader, if that is what the authors think.**

In the submitted manuscript, we have rewritten the key sentences in the abstract as follows: “The age- and gender-adjusted prevalence of trichiasis in ≥15-year-olds was 0.3% (95% CI 0.00-0.85), but no individual with trichiasis had trachomatous scarring (TS).”

**Abstract: The Abstract needs to reflect the high rate of access to water and sanitation, then state no relationship was found with the presence of TF, so the reader quickly has an understanding of why that might be.**

We have updated the abstract at line 71 to state “There were high rates of access to water and sanitation and no WASH variable was associated with the presence of TF”

**Line 89-90: Introduction: Again, the description of the pathogenesis of trichiasis is really for trachomatous trichiasis and should be labelled as such, not just trichiasis. Here would be an excellent place to indicate that while there are other causes of trichiasis, TT is the result of progressive trachomatous scarring.**

We have updated the introduction at line 98 to state “It is important to note that while there are other causes of trichiasis, TT is the result of progressive trachomatous scarring”

**Line 96: please change to “where prevalence of trachomatous trichiasis (TT) is >0.2%**

≥0.2% is correct, additional reference added to clarify.

**Line 124: I believe that one of the surveys in Peru collected all the indicators as well-if the authors mean the first national survey, because the entire nation was done in one survey, then they are likely right as very few countries are that small.**

We undertook further review of the literature and were unable to find any published study in Peru which assessed clinical, bacteriological, serological, and WASH markers of trachoma in children aged 1–9 years. However, our intention was to indicate the meaning that the reviewer suggests in the second half of their comment above. To avoid misinterpretation, we have changed “national” to “nationwide”.

**Line 194: Given that the infection rate is so high, higher than the rate of TF, and does not go up with age, suggests that there may have been field and/or lab contamination. Can the control procedures to avoid field contamination be described? Were control swabs taken to check for field contamination? The high rate of infection positivity in those without disease is further concerning.**

See detailed response above to comments about contamination.

**Line 223: how many plates were not acceptable. Were the samples rerun?**

We have updated the manuscript at line 259 to read: “Initially, 4 out of 21 plates (19%) did not meet quality control criteria provided by the CDC. The samples in these plates were re-punched, re-eluted and re-tested. Each repeat plate then passed quality control criteria.”

**Results and Figure 3. The data provide further supportive evidence of contamination. When fully 61% of positive test of infection is not accompanied by presence of disease, and half of those don’t even have positive serology, then one must investigate if contamination is an issue. We expect no disease but positive serology, given the cumulative nature of seropositivity-but that does not hold for infection**

See detailed response above to comments about contamination.

**Line 389. Discussion: The authors need to consider that the results reflect a problem with the infection data-if indeed there was contamination, and several infection negative children were misclassified, this could produce the result found.**

See detailed response above to comments about contamination.

**Line 398: the limitation section needs more serious thought. How about the absence of data on other factors that may reflect transmission environment, like household crowding? Other WASH indicators may have found an association like frequency of face or handwashing-hence the need to put table S1 in the paper and not in the supplemental tables. Although mentioned briefly, the high proportion of children with very good access to water and improved latrines does limit the ability to find any association, and the authors could provide power calculations to back that up. As for scarring, are there any images of the eyelids in adults that could be checked for presence of scarring. Scarring severe enough to produce TT is usually easily visible, so that could be backed up with images.**

Please refer to our earlier response to comments about limitations.

**I find that Table S1 is more valuable than table 1, which can be easily summarized in the text, if only a few tables/figures are allowed. Table S1 provides more detail on the WASH variables, and also shows the problem of trying to find associations when there is scant dispersion.**

We have included Table S1 into the manuscript text as Table 1.

REFERENCES

1.	Solomon AW, Holland MJ, Burton MJ, West SK, Alexander ND, Aguirre A, et al. Strategies for control of trachoma: observational study with quantitative PCR. The Lancet. 2003;362(9379):198-204. doi: 10.1016/S0140-6736(03)13909-8.

2.	Macleod CK, Butcher R, Javati S, Gwyn S, Jonduo M, Abdad MY, et al. Trachoma, anti-Pgp3 serology and ocular Chlamydia trachomatis infection in Papua New Guinea. Clinical Infectious Diseases. 2020. doi: 10.1093/cid/ciaa042.

3.	Cama A, Muller A, Taoaba R, Butcher RMR, Itibita I, Migchelsen SJ, et al. Prevalence of signs of trachoma, ocular Chlamydia trachomatis infection and antibodies to Pgp3 in residents of Kiritimati Island, Kiribati. PLoS Negl Trop Dis. 2017;11(9):e0005863. Epub 2017/09/13. doi: 10.1371/journal.pntd.0005863. PubMed PMID: 28898240; PubMed Central PMCID: PMCPMC5609772.

4.	Burr S, Hart J, Edwards T, Baldeh I, Bojang E, Harding - Esch E, et al. Association between Ocular Bacterial Carriage and Follicular Trachoma Following Mass Azithromycin Distribution in The Gambia. PLoS Negl Trop Dis. 2013;7(7). doi: 10.1371/journal.pntd.0002347.

5.	Macleod C, Yalen C, Butcher R, Mudaliar U, Natutusau K, Rainima-Qaniuci M, et al. Eyelash Epilation in the Absence of Trichiasis: Results of a Population-Based Prevalence Survey in the Western Division of Fiji. PLoS Negl Trop Dis. 2017;11(1):e0005277. Epub 2017/01/24. doi: 10.1371/journal.pntd.0005277. PubMed PMID: 28114364; PubMed Central PMCID: PMCPMC5256864.

6.	Butcher RM, Sokana O, Jack K, Macleod CK, Marks ME, Kalae E, et al. Low Prevalence of Conjunctival Infection with Chlamydia trachomatis in a Treatment-Naive Trachoma-Endemic Region of the Solomon Islands. PLoS Negl Trop Dis. 2016;10(9):e0004863. Epub 2016/09/08. doi: 10.1371/journal.pntd.0004863. PubMed PMID: 27603015; PubMed Central PMCID: PMCPMC5014345.

7.	Cocks N, Rainima-Qaniuci M, Yalen C, Macleod C, Nakolinivalu A, Migchelsen S, et al. Community seroprevalence survey for yaws and trachoma in the Western Division of Fiji. Trans R Soc Trop Med Hyg. 2016;110(10):582-7. Epub 2016/11/18. doi: 10.1093/trstmh/trw069. PubMed PMID: 27852877; PubMed Central PMCID: PMCPMC5155547.

8.	Macleod CK, Butcher R, Mudaliar U, Natutusau K, Pavluck AL, Willis R, et al. Low Prevalence of Ocular Chlamydia trachomatis Infection and Active Trachoma in the Western Division of Fiji. PLoS Negl Trop Dis. 2016;10(7):e0004798. Epub 2016/07/13. doi: 10.1371/journal.pntd.0004798. PubMed PMID: 27404379; PubMed Central PMCID: PMCPMC4942140.

9.	Butcher R, Sokana O, Jack K, Sui L, Russell C, Last A, et al. Clinical signs of trachoma are prevalent among Solomon Islanders who have no persistent markers of prior infection with Chlamydia trachomatis. Wellcome Open Res. 2018;3:14. doi: 10.12688/wellcomeopenres.13423.1.

10.	Butcher RMR, Sokana O, Jack K, Kalae E, Sui L, Russell C, et al. Active Trachoma Cases in the Solomon Islands Have Varied Polymicrobial Community Structures but Do Not Associate with Individual Non-Chlamydial Pathogens of the Eye. Front Med (Lausanne). 2018;4:251. Epub 2018/02/08. doi: 10.3389/fmed.2017.00251. PubMed PMID: 29410954; PubMed Central PMCID: PMCPMC5787070.

11.	Vasileva H, Butcher R, Pickering H, Sokana O, Jack K, Solomon A, et al. Conjunctival transcriptome profiling of Solomon Islanders with active trachoma in the absence of Chlamydia trachomatis infection. Parasites Vectors. 2018;11(1). doi: 10.1186/s13071-018-2682-2.

12.	Ministry of Health of Nauru. Nauru Global AIDS Progress Report. 2016.

13.	Mabey DCW, Solomon AW, Foster A. Trachoma. The Lancet. 2003;362(9379):223-9. doi: 10.1016/s0140-6736(03)13914-1.

14.	Solomon AW, Peeling RW, Foster A, Mabey DC. Diagnosis and assessment of trachoma. Clin Microbiol Rev. 2004;17(4):982-1011. Epub 2004/10/19. doi: 10.1128/CMR.17.4.982-1011.2004. PubMed PMID: 15489358; PubMed Central PMCID: PMCPMC523557.

15.	Butcher R, Handley B, Garae M, Taoaba R, Pickering H, Bong A, et al. Ocular Chlamydia trachomatis infection, anti-Pgp3 antibodies and conjunctival scarring in Vanuatu and Tarawa, Kiribati before antibiotic treatment for trachoma. Journal of Infection. 2020. doi: 10.1016/j.jinf.2020.01.015.
