# Supplementary material for: A national survey integrating clinical, laboratory, and WASH data to determine the typology of trachoma in Nauru
Source: PLoS Negl Trop Dis. 2022 Apr 19;16(4):e0010275. doi: 10.1371/journal.pntd.0010275 (PMC9017947; doi:10.1371/journal.pntd.0010275)
Supplement: Attachment — Submitted filename: Response to reviewers_08FEB22.docx [file pntd.0010275.s006.docx]

**RESPONSES TO REVIEW RECOMMENDATIONS**

**A national survey integrating clinical, laboratory, and WASH data to determine the typology of trachoma in Nauru**

Please note, line numbers used in this document refer to the tracked changes version of the manuscript.

**Thank you to the authors for their thoughtful responses to the Reviewers' first round of comments. After further review, there remain 2 main issues that are concerning the reviewers.**

**For the first point, about the possibility of contamination in the samples, the authors have been very clear in their explanation of contamination control during the laboratory work. For the possibility of field contamination, are the authors able to provide any more information as to how the samples were collected in the field, for example the training involved, and whether or not grader worked alone to do the work or whether there was assistance, possibly a "tube holder" if you will? The authors are recommended to either include the point in the limitation section as the reviewer suggests, or further evaluate/discuss the presence of high infection across the age groups, even among those without clinical signs for or serological responses and what that may mean in this population.**

We have provided additional detail in the manuscript to describe the team composition to make it clear that graders did not work alone and to detail the collection of samples in the field. As suggested, we have also further discussed the presence of high infection without clinical signs or seropositivity. These changes can be found at line:

- 165: “Each team consisted of a minimum of four individuals: a team leader, certified grader, certified recorder, and a DBS specimen collector”
- 208: “Conjunctival specimens were collected by certified graders with assistance for specimen collection provided by the team leader and DBS specimen collector. The assistants were responsible for tube holding and specimen labelling. In the week prior to the survey, the team undertook training in conjunctival and DBS sample collection. This training incorporated infection control procedures to minimise risk of specimen contamination in the field. For the first three days of fieldwork, specimen collection was supervised by a senior investigator.”
- 421: “While there are individuals who are PCR positive without TF or seropositivity for chlamydial antibodies, generally these outcomes co-occur within individuals. PCR positivity in the absence of TF or seropositivity was more common in younger children, possibly reflecting individuals with early infections who did not yet have inflammatory sequalae resulting in clinical signs and without an exposure level sufficient for seroconversion.”

**For the second point on the grading of TS, while they may not have "standardized" their TS grading, the authors are clear in their responses that they trust their graders. To increase trust among readers, the authors need to further detail the Tropical Data training that they used as it relates to training on TT and TS specifically, including what classroom and/or field training went into the assessment of TT and TS. The authors are further welcome to recommend whether they think enhanced training and/or data collection would have been useful.**

We have provided additional details in the manuscript to address this feedback. These can be found at the following lines:

- 188: “In the week prior to the survey, field graders undertook a four-day training programme conducted by certified Tropical Data trainers using standardised training material. During the classroom session on examination techniques, trainees were taught how to recognise trichiasis (upper and lower lid) and TS. Consistent with the Tropical Data collection method, trainees were taught to recognise and collect data on only moderate or severe scarring as per the WHO simplified grading system definition of TS.”
- 491: “As we approach global elimination more multi-modal, integrated screening and research, like this work, will be required. Given this, it would be ideal to have standardised methods for such work covering the capture, reporting, and interpretation of clinical, infection, and serosurvey data. Standardisation would allow for comparability across locations and provide a layer of quality control for reported results. Without such standardization, the assessment of scarring is difficult to interpret, and it is possible that cases of trichiasis are due to trachoma.”
